# Factors affecting intraosseous pressure measurement

**DOI:** 10.1186/s13018-018-0877-z

**Published:** 2018-07-28

**Authors:** Michael Beverly, David Murray

**Affiliations:** Botnar Research Centre, Nuffield Department of Orthopaedics, Rheumatology & Musculoskeletal Sciences, University of Oxford, Nuffield Orthopaedic Centre, Headington, Oxford, OX3 7LD UK

**Keywords:** Intraosseous pressure, Physiology, Subchondral, Bone blood flow, Vascular occlusion, Osteoarthritis, Osteonecrosis

## Abstract

**Background:**

Although a raised intraosseous pressure (IOP) has been found in osteoarthritis and osteonecrosis, the normal physiology of subchondral circulation is poorly understood. We developed an animal model and explored the physiology of normal subchondral perfusion and IOP.

**Methods:**

In 21 anaesthetised rabbits, 44 intraosseous needles were placed in the subchondral bone of the femoral head (*n* = 6), femoral condyle (*n* = 7) or proximal tibia (*n* = 31). Needles were connected to pressure transducers and a chart recorder. In 14 subjects, the proximal femoral artery and vein were clamped alternately. In five subjects, arterial pressure was measured simultaneously in the opposite femoral artery.

**Results:**

The average IOP at all 44 sites was 24.5 mmHg with variability within SD 6.8 and between subjects SD 11.5. IOP was not significantly influenced by gender, weight, site or size of a needle. Needle clearance by flushing caused a prolonged drop in IOP whereas after clearance by aspiration, recovery was rapid. IOP recordings exhibited wave patterns synchronous with the arterial pulse, with respiration and with drug circulation time. There was a correlation between IOP and blood pressure (13 sites in 5 subjects, Pearson correlation 0.829, *p* < 0.0005). There was a correlation between IOP and the associated pulse pressure (PP) in 44 sites among 21 subjects (Pearson correlation 0.788, *p* < 0.001). In 14 subjects (31 sites), arterial occlusion caused a significant reduction in IOP and loss of PP (*p* < 0.0001). Venous occlusion significantly raised IOP with preservation of the PP (*p* < 0.012).

**Conclusion:**

Our study shows that subchondral cancellous bone behaves as a perfused tissue and that IOP is mainly a reflection of arterial supply. A single measure of IOP is variable and reflects only perfusion at the needle tip rather than being a measure of venous back pressure. Alternate proximal vessel clamping offers a new means of exploring the physiology of subchondral perfusion. We describe a model that will allow further study of IOP such as during loading.

## Background

Intraosseous pressure (IOP) and bone blood flow have been studied by authors interested in bone circulation and physiology for more than 50 years. Varying techniques using different needles, flushing and recording methods have produced differing results, and there has been limited progress in understanding IOP physiology since Azuma reported IOP fluctuation in a rabbit model in 1964 [1–7]. Measurement of IOP in man has also proved variable [8]. IOP has generally been found to be raised in bone diseases such as osteonecrosis and after steroid use [9–13]. A raised IOP has been associated with pain in osteoarthritic joints, chondromalacia patellae and with cartilage degeneration. IOP may also be important in driving fluid through canaliculi, hence governing osteocyte activity and bone turnover [14, 15]. It has usually been thought that IOP had a static or fixed value which was due to venous back pressure, intramedullary pressure or interstitial pressure [2]. IOP has been measured experimentally in animals and in man. Steroid-induced models of avascular necrosis have been developed in order to study IOP and its treatment [16, 17]. Ficat and others developed a technique for the ‘functional exploration’ of bone in patients with early osteonecrosis [10]. However, the factors that control IOP at rest and during activity and the physiology of subchondral bone circulation remain largely unknown [18].

In a preliminary unpublished clinical work, we measured IOP prior to forage, osteotomy or decompression. The findings were variable. The rationale for our study was to explore the physiology of IOP in healthy subchondral cancellous bone at rest in an animal model.

## Methods

### Experimental measurements

**Fig. 1:**
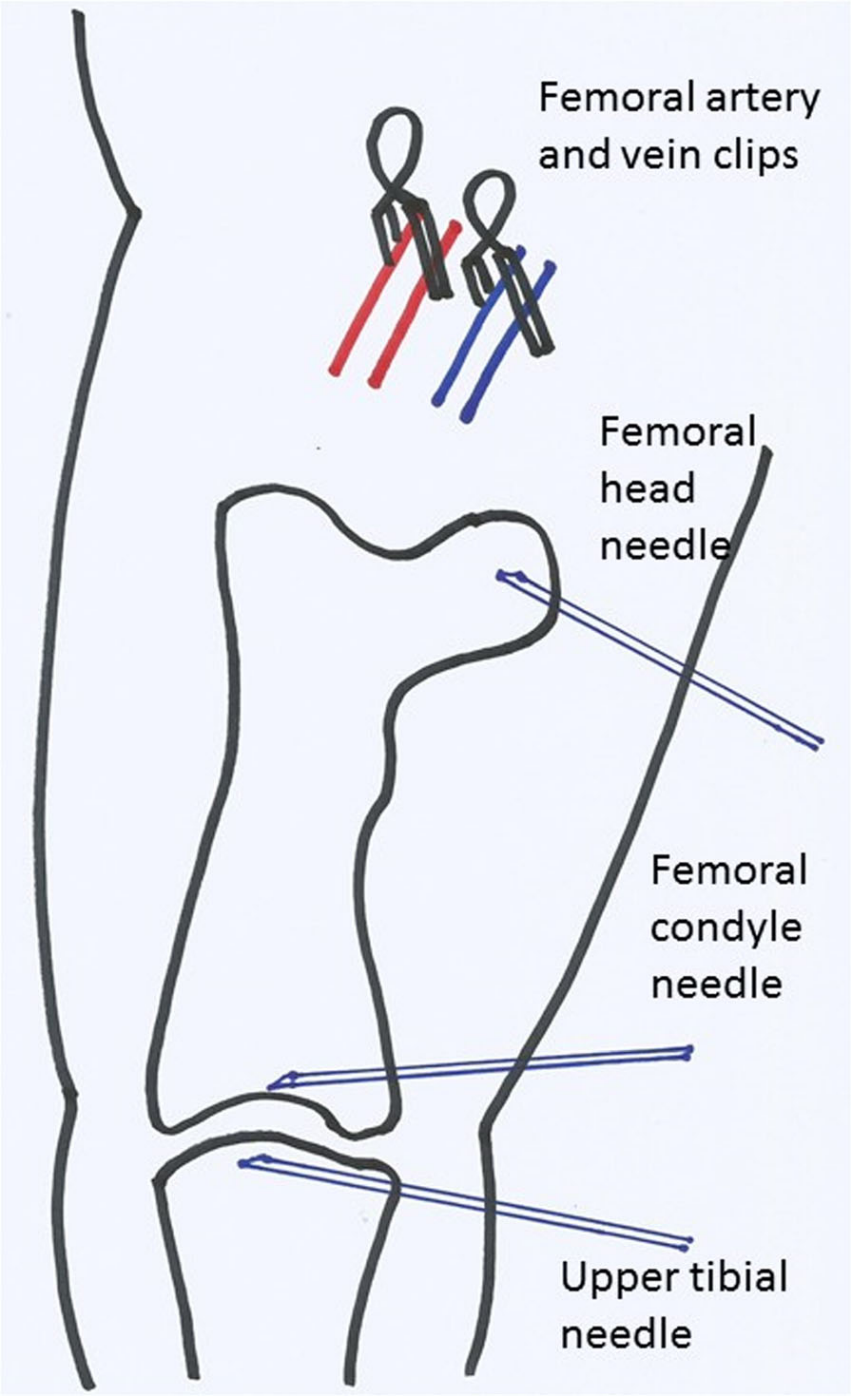
Experimental set up. Illustration showing position of vascular clips and intraosseous needles

IOP was measured experimentally using intraosseous needles in the subchondral cancellous bone of the femoral head, femoral condyle and proximal tibia of 21 adult New Zealand White rabbits (Royal Postgraduate Medical School, Home Office licence ELA 24/4994). The rabbits were anaesthetized through an ear vein with intravenous Sublimaze (fentanyl, Janssen-Cilag Pty Ltd., NSW 2113, Australia) and Valium (diazepam, Genentech Inc., San Francisco, CA 94080, USA). Induction of anaesthesia was by IV Sublimaze (fentanyl) 2 ml of 0.05 mg/ml solution depending on the size of the animal with top up IV infusion of Valium (diazepam) 0.5 ml of 5 mg/ml solution alternating with Sublimaze (fentanyl) 0.5–1.0 ml given slowly on approximately a ½ hourly basis. The initial recordings were mainly from the right femoral head with later recordings mainly from the femoral condyles and proximal tibia as in Fig. 1 as these were more reliable. There were 44 different IOP recordings, six at the femoral head, seven from the femoral condyles and 31 from the proximal tibiae. The femoral head was approached by dissection anteriorly through the femoral triangle. A saline-filled 21G (*n* = 13) or 23G (*n* = 31) venesection needle was pushed by hand into the femoral head subchondral bone by rocking the needle through a 5° to 10° arc along the line of the bevel of the needle. The needles were placed under direct vision in the approximate centre of the subchondral region within a few millimetres of the subchondral surface. For the femoral condyle, insertion was a few millimetres proximal to the visible and palpable surface. The same was applied to the proximal tibia. In 14 subjects, the right femoral artery and vein were selectively clamped at the inguinal ligament. The needles were connected by saline-filled lines to pressure transducers (Druck PDCR75, Druck Ltd., Leicester LE6 0FH, UK) and to a four-channel chart recorder (Lectromed MX4P-31, Lectromed Ltd., Jersey, Channel Islands, UK). The transducers were calibrated on a 0–100 mmHg scale. In five animals, the arterial blood pressure was measured by cannulating the left femoral artery and recording pressure using the same transducer system.

The experimental set up was as illustrated in Fig. 1.

### Statistical analysis

Experimental time varied from about 15 min to over 2 h. Intraosseous pressure and the associated pulse pressure (PP) were measured at all 44 sites on three occasions, early, middle and late during each experiment and were averaged. Results were expressed as means, standard deviations and ranges. Student’s *t* test was used to determine if there were significant differences. When each subject was used as its own control, paired tests were used. Otherwise, unpaired tests were used. The Pearson test was used to assess correlations. *P* < 0.05 was considered to be statistically significant.

### Experimental plan

A series of experiments was undertaken to investigate the following:

1. Normal baseline IOP: The normal IOP was measured at all 44 sites and the influence of factors relating to animal, including gender, weight, site and size of needle were explored.
2. Method of measuring: The Ficat method in which a 0.5 ml clearance bolus of saline was injected was compared with a method using needle clearance by aspiration [10].
3. IOP wave forms: IOP wave forms were explored at different chart speeds, 12.5 mm/sec and 12.5 mm/min. The traces were compared with the directly visible pulse and respiratory waves. The chart speed was 12.5 mm/minute in subsequent experiments.
4. Drug administration: The effect of a bolus drug administration of Valium (diazepam 2.5 mg) or Sublimaze (fentanyl 0.05 mg) on IOP was recorded.
5. Relationship between systemic blood pressure, IOP and the pulse pressure (PP)
  The relationship between systemic blood pressure and IOP in 13 sites among 5 subjects was explored.
6. The relationship between IOP and pulse pressure: The relationship between IOP and the associated pulse pressure was explored at 44 sites among all 21 subjects. The pulse pressure was taken as the difference between the top and bottom of the wave on the IOP tracing at that point.
7. Vascular occlusion: The effect on IOP of clamping the femoral artery or femoral vein was recorded in 31 sites among 14 subjects. The clamps were small ‘bulldog’ sheathed vascular clips. They were applied to the proximal femoral artery or vein under direct vision.

## Results

### Normal IOP

**Fig. 2:**
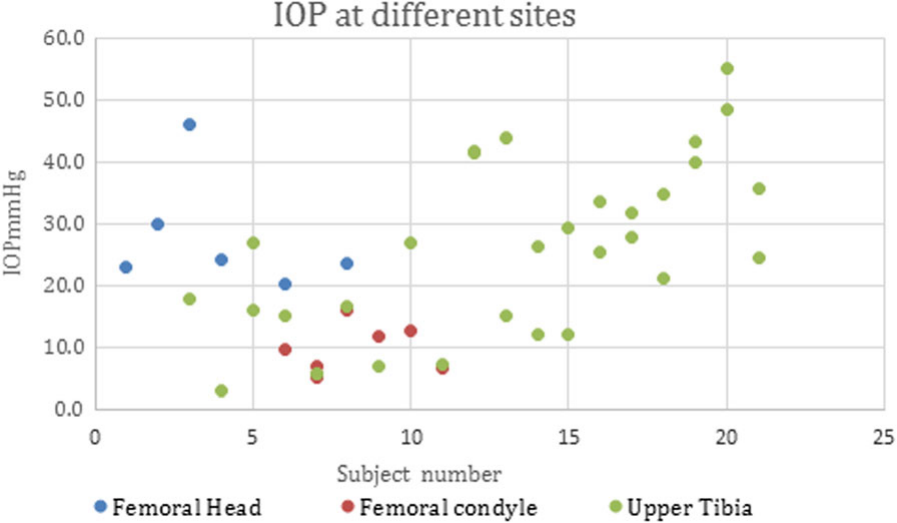
Variability in IOP. Graph showing variation in IOP between subjects (mean 24.5 mmHg, SD 11.5) and within subjects (mean 25.9 mmHg, SD 6.8) in 21 subjects at 44 different sites

Basal intraosseous pressure was measured at 44 sites among 21 subjects as in Fig. 2. At each site, the early, middle and late IOP and the corresponding PP were averaged. The averaged IOP at different sites varied considerably (mean 23.4 mmHg, SD 13.7, range 3–55). Gender (female *n* = 14, male *n* = 7) had no effect on IOP (*t* test *p* = 0.537). Weight (2920–5560 g) had no effect on IOP (Pearson correlation *p* = 0.368). Needle size 21G (*n* = 13) and 23G (*n* = 31), *t* test 0.14, had no effect on IOP.

Several subjects had two or more sites measured. There was variation between subjects (SD 11.5 mmHg) and variation between sites within subjects (SD 6.8 mmHg). Where paired measurements were available (*n* = 7), there was no difference (*t* test *p* = 0.52) between the femoral condyle (mean 9.8, SD 3.6, range 5–16) and proximal tibia (mean 12, SD 7.4, range 5.7–16.7). There were insufficient paired data for femoral head comparisons.

### Method of measurement of IOP

**Fig. 3:**
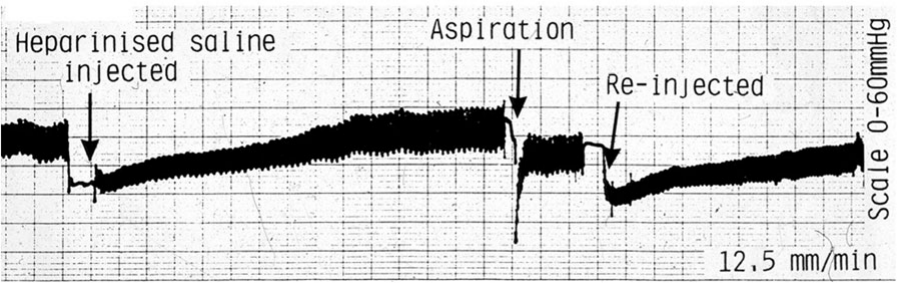
The effect of saline injection and aspiration. A representative trace from a typical experiment showing the effect of a 0.5 ml saline bolus flush injection with about 8 min to recover followed by aspiration with recovery in less than 30 s. A second bolus flush injection and slow recovery follows. Large square = 0.5 cm

Previous investigators have used a saline bolus or ‘clearance’ injection [10] before recording IOP. We found that in healthy bone this caused a marked fall in IOP which was followed by a gradual recovery taking up to 10 min. Aspiration was followed by faster recovery which took less than 1 min as in Fig. 3.

### IOP wave forms

**Fig. 4:**
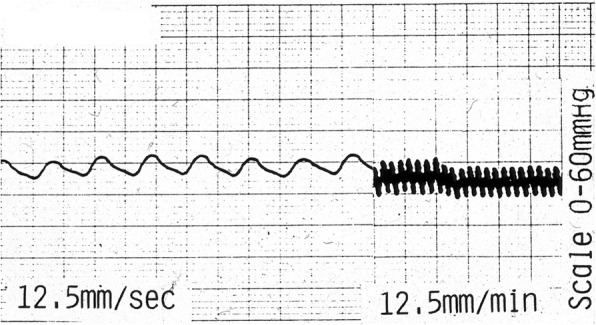
Cardiac and respiratory waves. A representative IOP recording at different speeds. On the left (12.5 mm/sec, eight pulses in 6 s, pulse rate 80/min), a wave which was seen to be synchronous with the arterial pulse is observed. On the right (at a speed 60 times slower (12.5 mm/min), for 3 min, eight respirations per minute), an underlying wave which was seen to be synchronous with respiration was observed. Large squares are 0.5 cm

The majority of recordings showed a pulsatile wave form. At a fast chart speed of 12.5 mm/sec, the wave was seen to be synchronous with the cardiac arterial pulse by direct observation. At a speed 60 times slower or 12.5 mm/min, the arterial pulse was obliterated but an underlying slower wave form was observed. This wave was seen to be synchronous with respiration by direct observation as in Fig. 4.

### Drug administration

**Fig. 5:**
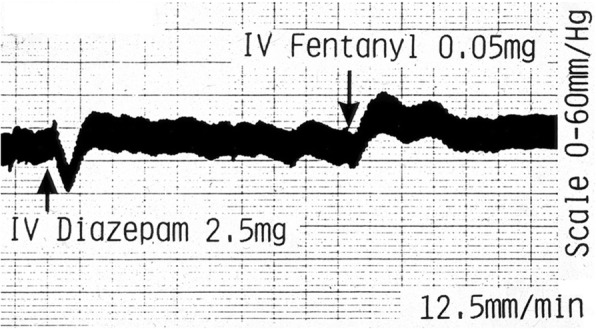
Effect of systemic drug administration. Systemic administration of anaesthetic agents such as diazepam and fentanyl affected the IOP as in this representative trace. Large square = 0.5 cm

Systemic administration of anaesthetic agents affected the IOP. For example, in the representative trace shown, the administration of an intravenous bolus of Valium (diazepam 2.5 mg) lowered the IOP and PP waves for about 90 s. Sublimaze (fentanyl 0.05 mg) caused a variable effect. In this representative trace, there was an excitatory effect. The IOP and PP rose for about 90 s with another lesser peak following. This double-wave pattern corresponds to the expected circulation time of the drug bolus as in Fig. 5.

### Relationship between IOP and blood pressure and IOP and PP

**Fig. 6:**
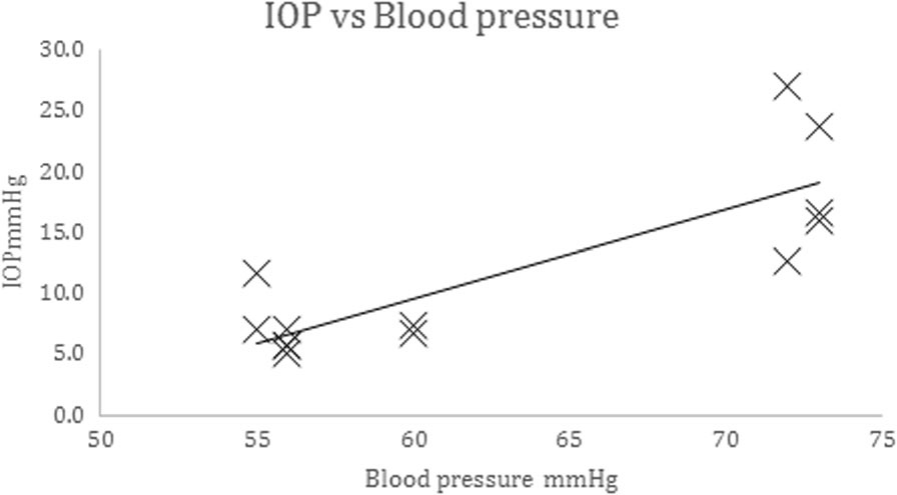
Correlation of IOP with blood pressure. Effect of blood pressure on IOP. Intraosseous pressure was measured at 13 sites among 5 subjects

Arterial blood pressure (BP) was measured in the left femoral artery in five animals using a pressure transducer. The mean BP was 62.8 mmHg, SD 8.2, range 55–73. Intraosseous pressure was measured at the same time at 13 different sites among the five subjects. The mean IOP was 11.7 mmHg, SD 7.2, range 5–27. There was a significant relationship (*p* < 0.0005) between blood pressure and IOP, Pearson correlation 0.829 as in Fig. 6.

### Relationship between IOP and pulse pressure

**Fig. 7:**
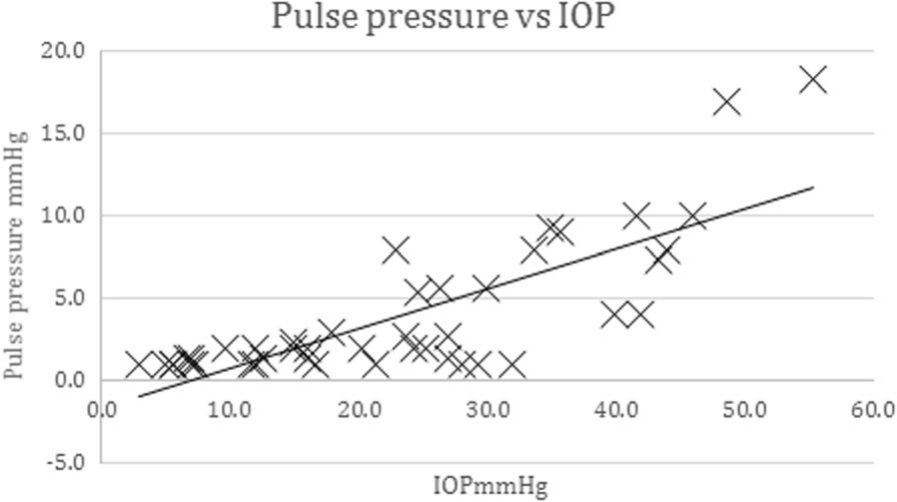
Correlation of pulse pressure with IOP. Pulse pressure (mmHg) with IOP (mmHg) for 44 separate sites among 21 subjects

IOP was compared with the associated pulse pressure (PP) at all sites (*n* = 44) among the 21 subjects. The pulse pressure was taken as the difference between the top and bottom of the IOP trace wave. There was a significant correlation (*p* < 0.0001) between IOP and the associated PP, Pearson correlation 0.788 as in Fig. 7.

### Effect of clamping supplying artery or draining vein

**Fig. 8:**
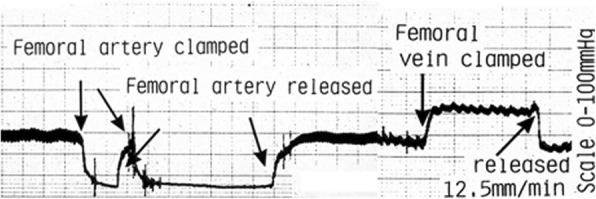
Example of arterial and venous occlusion. Recording showing the effect of clamping and releasing the proximal femoral artery (left half) and clamping and releasing the proximal femoral vein (right side). On the left side, the arterial clamped slipped and was re-applied. Large square = 0.5 cm

In 14 subjects (31 sites), when the proximal femoral artery was clamped at the inguinal ligament, there was a fall in IOP with loss of the pulse pressure immediately. IOP stabilised within about 1 min as in Fig. 8. Recovery in IOP and restoration of the pulse pressure wave following release of the arterial clip was seen after less than 1 min. When the proximal femoral vein was clamped, the IOP rose and the pulse pressure was preserved as in Fig. 8. With clamping the proximal femoral vein, there was a pressure rise taking up to 30 s to stabilise and a similar time after venous release before the IOP returned to normal. During proximal venous clamping, the pulse pressure or respiratory wave was preserved. The period during which the clamp was applied was typically 3–5 min as shown in the representative traces in Fig. 8.

**Fig. 9:**
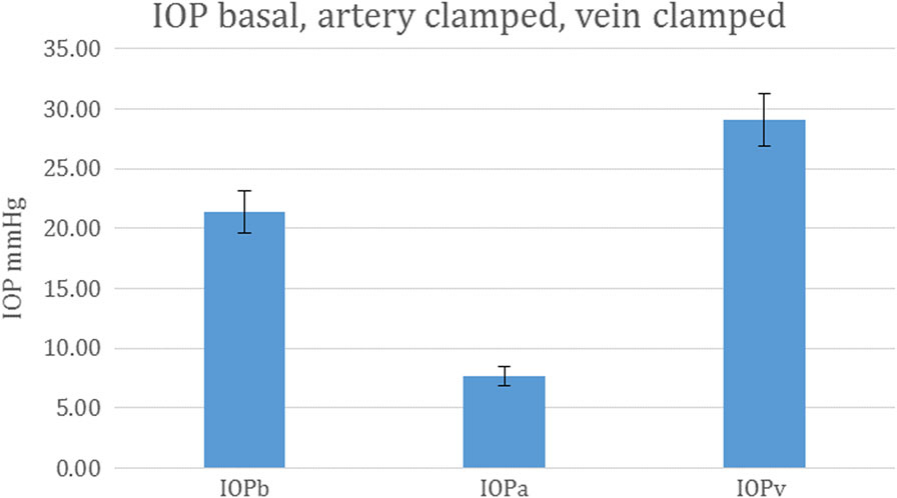
Effect of arterial and venous occlusion. Effect of vascular occlusion on IOP at 31 sites in 14 subjects. Error bars are SEM. Each subject acted as their own control

For 31 sites among the 14 subjects, the mean basal IOP (IOPb) was 20.4 mmHg (SD 13.5 mmHg). The mean arterial occlusion IOP (IOPa) was 7.5 mmHg (SD 4.6 mmHg), and the mean venous occluded IOP (IOPv) was 28.8 mmHg (SD 12.0 mmHg). We compared IOPb vs IOPa and IOPv using paired *t* tests. Each subject was its own control. There were significant differences between them, IOPb vs IOPa *p* < 0.00001, IOPb vs IOPv *p* < 0.012, and IOPa vs IOPv *p* < 0.00001 as shown in Fig. 9.

## Discussion

This study of the physiology of resting intraosseous pressure measurement in a healthy animal model gives insights which contradict some current views. IOP has usually been thought to have a fixed or static value which reflects an interstitial pressure, tissue turgor or venous back pressure [2, 10]. We found variation in IOP between and within normal subjects even when using a standardised approach. Although IOP was not related to gender, weight, size or site of needle, it was proportional to blood pressure. In addition, pulse pressure was proportional to IOP. The demonstration of variability in IOP and proportionality between IOP and PP shown by our 44 different sites in otherwise normal bone strongly suggests that the needle tip strikes different vessels within the cancellous bone by chance and that the IOP and PP reflect conditions in the blood pool at the needle tip rather than IOP being of a fixed value for any whole bone or subject. In the blood pool at the needle tip, we assume that IOP from the highest pressure vessel will be recorded. Larger arterioles will give a higher IOP and PP as they are nearer the arterial side of the vascular tree. Smaller vessels or capillaries give a lower IOP and lower PP. Venules, fat and trabeculae may return virtually no pressure.

IOP not only correlated with pulse pressure, but IOP also correlated with blood pressure, and was seen to correspond to respiratory waves and systemic or drug circulatory effects further suggesting that IOP is governed mainly by the arterial circulation. Moreover, there was a greater fall in IOP with arterial occlusion than a rise with venous occlusion. This would also indicate that the majority of the recorded IOP is due to the arterial supply side. The disappearance from the trace of the pulse pressure with arterial occlusion also demonstrates that the IOP and both the associated pulse and respiratory waves are mediated through the arterial supply. Both are preserved when the proximal femoral vein is clamped. Once again this suggests that IOP is therefore a ‘supply’ side phenomenon rather than a venous back pressure or measure of drainage. We found no previous references to this analysis of the physiology of IOP [8].

We have shown that IOP is not a constant but varies between and within subjects and also depends on the method and timing of measurement. Within that variability, there are recognisable wave forms with arterial, respiratory and drug or circulatory time patterns.

Whilst trying to optimise the method of measuring IOP, we compared the usual standard method, which involves injecting a small clearance bolus of saline (Ficat technique), with a novel method in which the needle was aspirated prior to IOP measurement. In healthy bone, both techniques resulted in an initial decrease in IOP which recovered but not necessarily to exactly the same starting point (Fig. 2). The recovery after aspiration was quicker and was usually stable within 1 min. After saline injection up to 10 min was required for recovery. Saline bolus injection appears to be harmful to the local microcirculation [19]. The delay in return to normal IOP may reflect a washout or recovery period. The subsequent slightly higher IOP may reflect renewed contact with larger arterial supply vessels following local destruction. The use of forced saline injection should probably be avoided. The aspiration method appears preferable. Previous workers assessing IOP after using the bolus injection method may in fact have been measuring an IOP which was temporarily lowered as a result of their saline injection [2, 7, 9, 10]. It is equally possible that in osteonecrotic bone the IOP recorded would be high because of the pressure of the injection itself into poorly drained or ischaemic bone. A third possibility is that the flushing of heparinised saline, blood, fat and bone fragments backwards into the delicate subchondral vascular tree is physically damaging or toxic [19]. The slower recovery after injection may represent a prolonged washout or recovery phase which is not required after less damaging aspiration.

The residual pressure after arterial clamping, for example in Fig. 8, probably does represent a real residual venous or tissue back pressure at the needle tip. Similarly with proximal venous occlusion, the IOP recorded may represent the best obtainable arterial supply pressure at that needle tip. For the first time, this simple approach gives a means of assessing the perfusion pressure range obtainable at the needle tip deep in the cancellous bone.

There are several potential limitations in this study. X-rays were not available for needle placement, but under direct vision hand placement of the needle tip was to within a few millimetres of the subchondral surface as illustrated in Fig. 1. The animals used were similar healthy adults but were not identical. Different sites were used, initially mainly the femoral head and later mainly at the femoral condyle and proximal tibia which were technically easier. We could not always obtain tracings from all sites in all subjects. The subject’s blood pressures inevitably varied during the experiments. Needle insertion could never be identical at all sites. The experiments varied in duration from about 15 min to over 2 h. Because IOP inevitably fluctuated during that time, an average of the early, middle and late values was used for each of the 44 sites. There was a learning curve but generally anaesthetic control and experimental duration increased with experience [7, 20, 21]. Although the known experimental differences resulted in some variability in results, our experimental conditions were generally representative of the clinical situation. The analysis was based on data from different sites. It could be argued that these are not completely independent as in some subjects there were two or three sites. However, the IOP was not significantly different at different anatomical sites. If the analysis was based on subjects alone, the numbers would be smaller. The work was primarily qualitative and designed to develop a standardised model. Nevertheless, some useful insights on perfusion physiology have been derived from our study. This may in turn allow better use of IOP measurement in the study of circulation in diseased bone states such as osteoarthritis and avascular necrosis. Future work will measure IOP under load and with vascular occlusion to give a better understanding of subchondral perfusion physiology.

## Conclusion

In conclusion, we have developed a model for IOP study. Flushing of saline and other material into the bone is probably harmful and causes a prolonged drop in IOP [19]. Aspiration clearance is likely to be better. Our work shows that IOP at rest in vivo mainly reflects physiological arterial pressure at the needle tip in a perfused organ. Whilst a single isolated measure of IOP is therefore of little value even in a standardised model, by alternate proximal arterial and venous clamping, a more useful idea of circulation at the needle tip deep in cancellous bone may be achieved.

## Abbreviations

IOP: Intraosseous pressure
PP: Pulse pressure
